# Secondary Myelitis in Dermal Sinus Causing Paraplegia in a Child with Previously Normal Neurological Function

**DOI:** 10.1155/2016/8918954

**Published:** 2016-12-06

**Authors:** Sakina Rashid, Grace Kinabo, Marissa Kellogg, William P. Howlett, Marieke C. J. Dekker

**Affiliations:** ^1^Department of Pediatrics, Kilimanjaro Christian Medical Centre, Moshi, Tanzania; ^2^Stanford University, Palo Alto, CA, USA; ^3^Department of Internal Medicine, Kilimanjaro Christian Medical Centre, Moshi, Tanzania; ^4^Radboud University Medical Centre, Nijmegen, Netherlands

## Abstract

Neural tube defects result from failure of neural tube fusion during early embryogenesis, the fourth week after conception. The spectrum of severity is not uniform across the various forms of this congenital anomaly as certain presentations are not compatible with extrauterine life (anencephaly) while, on the other hand, other defects may remain undiagnosed as they are entirely asymptomatic (occult spina bifida). We report a child with previously normal neurological development, a devastating clinical course following superinfection of a subtle spina bifida defect which resulted in a flaccid paralysis below the level of the lesion and permanent neurological deficits following resolution of the acute infection and a back closure surgery.

## 1. Introduction

Spina bifida occulta is the most benign presentation of a neural tube defect in newborns. Based on CT scan findings of 228 paediatric patients and 235 adults who underwent abdominal and pelvic imaging for reasons unrelated to the spine, spina bifida occulta seems to be subclinically present in a sizeable proportion of screened children (41.2%) with a much lower figure reported for adults (7.7%) [1]. It should be stressed that the abnormality is often subclinical and the significant fall in prevalence may indicate that complete fusion of the vertebral arches occurs beyond childhood in a significant number of individuals. The cutaneous manifestations of this defect shows extensive variability, ranging from normal appearance, to hemangioma, skin discolouration, hairy patch, pit, dermal sinus, or nodule [2]. The defect typically occurs at the L5 and S1 vertebral levels.

## 2. Methods

This clinical case study was approved of by the Medical Ethics Committee of Kilimanjaro Christian Medical Centre, Moshi, United Republic of Tanzania. Informed written consent was obtained from the child's mother.

## 3. Case Presentation

### 3.1. History

A two-year-old Tanzanian girl presented with a 1-week history of fevers up to 38.5°C, flaccid lower limb paralysis, and loss of sphincter control. Prior to admission, neurological development had been normal with good sphincter control. Her mother had noticed a small reddish pimple on her back since birth, which had never discharged fluid or grown in size.

### 3.2. Examination

On examination, the child was febrile with normal level of consciousness, meeting cognitive milestones, normal neurological examination above the waist, and flaccid paralysis of the legs. There appeared to be decreased sensation roughly from the umbilicus down. No structural abnormalities of the lower extremities were appreciated: leg lengths were equal, without atrophy or foot deformity. A soft lipomatous midline swelling of 2 by 2 cm was observed at a low-thoracic level, which had a minute central pore without discharge. Spinous processes could not be palpated in the region surrounding the lesion. Mild thoracolumbar scoliosis and mildly increased lumbar lordosis were observed. However, the child was unable to sit unassisted. Over the course of the admission, she had a single generalised seizure with quick recovery of consciousness and transiently complained of a painful feeling in her right arm. Due to the young age, the postulated meningitis with the present sinus, and seizures in the course of admission to the hospital, we assumed that the pain which the child consistently indicated in the right arm may have a neuropathic origin, for which we prescribed low-dosage amitriptyline.

### 3.3. Differential Diagnosis

The working diagnosis was superinfection of a dermal sinus from a spina bifida occulta defect in a previously neurologically normal child, leading to spinal cord and/or nerve root compromise and bacterial meningitis. The lower extremity paralysis and apparent sensory level were evidence of spinal cord and/or nerve root compromise, either by direct myelitis/polyradiculitis or by compression from epidural abscess formation. The episode of seizures and the unilateral cervical radiculitis was evidence of a component of meningitis. Alternative diagnoses on the differential included: common bacterial meningitis with secondary involvement of the spinal cord, an inflammatory or infectious polyradiculitis, or thoracic spine Pott's disease with secondary paraplegia.

### 3.4. Additional Examinations

A lumbar puncture could not be performed because of the midline lumbar defect and the suspected infected dermal sinus. Initial lumbar X-ray (limited by underexposure) showed no evidence of vertebral displacement or collapse. An ultrasound examination of the swelling identified a superficial cyst, without clear connection to the spinal column. A Full Blood Picture (FBP) demonstrated an elevated white cell count with a predominance of lymphocytes (see Table 1).

### 3.5. Clinical Course

The child was treated with broad-spectrum intravenous antibiotics, ceftriaxone, cloxacillin, and metronidazole, to cover for aerobic and anaerobic causes of bacterial meningitis and abscess. Unfortunately, blood cultures had not been done prior to starting empirical antibiotic therapy. Five weeks after admission, the child and her mother absconded from the hospital due to inability to afford the cost of care. The family was also unable to afford a spinal CT scan (75 American Dollars) during admission.

The child and her mother returned to the paediatric neurology clinic for a follow-up examination 10 months later, having managed to afford CT spine. The two-centimetre midline nodule had subsided; only a two-millimetre papule with a small central invagination remained in its place. Over two years, the child's legs had become spastic, left more than right, and she developed persistent urinary and faecal incontinence. She maintained a sense of bladder fullness which facilitated bladder catheterisation. Progressive scoliosis with marked lumbar lordosis was noted (see Figure 3).

**Figure 1:**
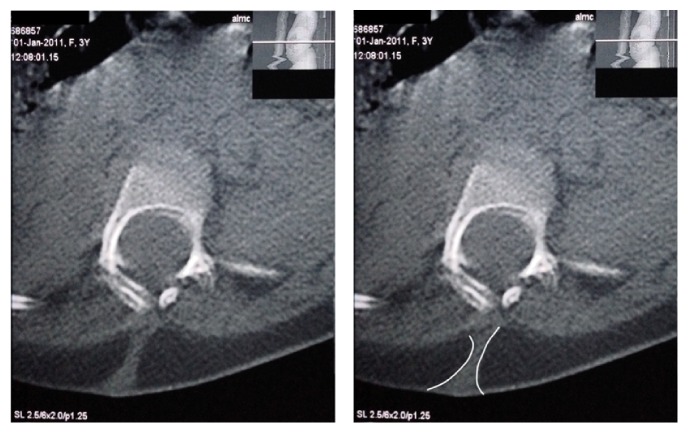
A transverse CT section demonstrating a dorsal dermal sinus connecting the epidermis to the epidural space.

**Figure 2:**
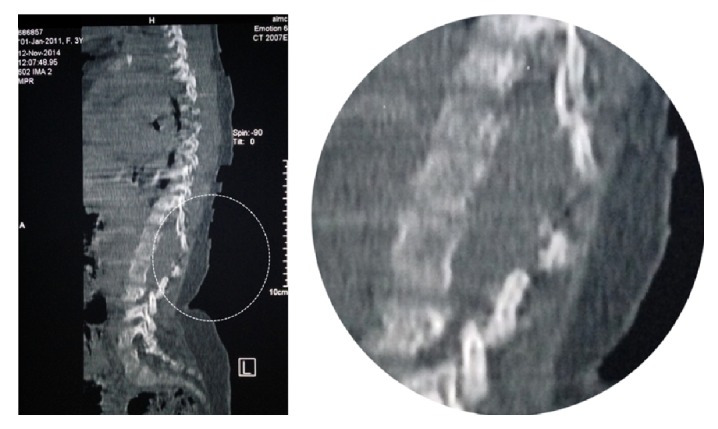
A sagittal CT section demonstrating a multiple level spinal dysraphism and deformity.

**Figure 3:**
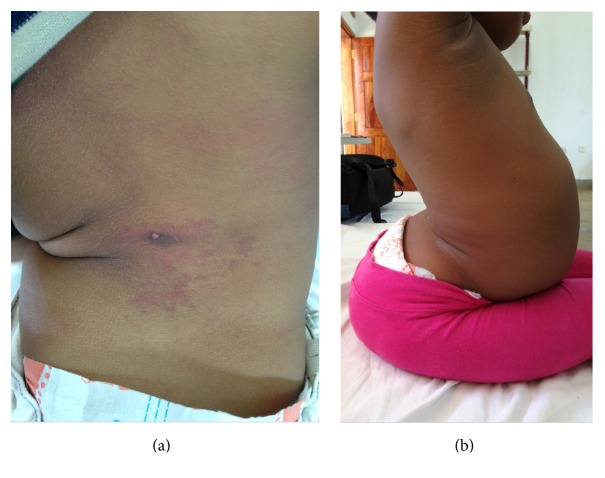
Physical examination demonstrated (a) the midline papule, a thoracolumbar scoliosis, and (b) marked lumbar scoliosis and lumbar lordosis.

The CT scan demonstrated multilevel spinal dysraphism and deformity above and below the level of the now-resolved swelling. Axial CT sections revealed a dorsal dermal sinus at the level of T12 connecting the epidermis to the epidural space. (See Figures 1 and 2 for the original axial and longitudinal sections as well as an enhanced representation of the abnormality.)

The dermal sinus caused recurrent bouts of fever with purulent discharge from the lesion. She was enrolled in a NGO-funded surgery program performing back closure operations for newborns with spina bifida. Since the surgical intervention was a free treatment option offered to the mother in the framework of a spina bifida NGO care program, extensive surgical reports were not made available for review. Following the surgery, the local discharge and bouts of fever resolved and, subsequently, her general condition improved. However, her neurological deficits remained unchanged.

## 4. Discussion

We present a child with previously normal neurological development and a devastating clinical course following rare superinfection of a subtle spina bifida defect. The diagnosis was evident upon visual inspection of the lesion and knowledge of spinal embryology. However, nearly one year passed before the family could afford imaging to confirm the clinical suspicion and even longer before the defect could be surgically repaired. Unfortunately, delay in diagnosis and treatment is common in rural Tanzania where the vast majority of individuals lack health insurance. The child's mother ran away from the hospital because she was unable to pay the bills. As a result, there had been progressive episodes of clinical infection and irreversible neurological damage. Moreover, it is important to note that spina bifida incidence is significantly reduced by adequate maternal prenatal folate intake. It is possible the defect could have been prevented entirely if the mother had access to prenatal care and adequate nutrition or dietary supplementation.

In this case study, the child had a dorsal dermal sinus, which is an epithelium-lined tract beginning at the epidermis and extending to variable depths into underlying structures [3]. Reported incidence is one in every 2500 live births [3]. The deformity results from abnormal neurulation during the fourth week of embryonic development; the neuroectoderm fails to separate completely from the surface ectoderm, and thus the mesodermal elements which normally surround the neural tube fail to assume their normal distribution [4]. The sinus may terminate in the subcutaneous levels and remain asymptomatic for life but in a certain subset of patients it terminates at the subdural level.

This manifestation of a neural tube defect is most likely to present at the lumbar and lumbosacral levels of the vertebrae with the cervical region involved in less than 1% of cases [4]. Rarely, a sinus may become infected and the likelihood that there will be concurrent meningitis and the presence of neurological signs will depend on whether the termination of the lumen is at an intraspinal level. While increasing depth of the sinus confers increasing risk for CNS infection, sinus infection can lead to abscess formation which can cause further invagination by infectious processes.

Patients with this inconspicuous malformation often come to medical attention with clinical complaints concerning sites of the body below the level of the lesion. Systemic reviews note that the presenting complaints primarily involve neurological or cutaneous abnormalities, signs of infection, urological or bowel abnormalities, and skeletal abnormalities; examples include scoliosis, limb weakness, muscle atrophy, gait disturbances, and congenital talipes equinovarus [5, 6]. In rural Tanzania, a small midline cutaneous abnormality or fever in a normally developing child would be unlikely reasons to seek formal medical attention. Therefore, the child in this case presented only after neurological deficits occurred.

Since a dermal sinus tract which terminates intrathecally is high-risk for complications of CNS infection, medical consensus is that lesions above the sacrococcygeal level be explored to the terminus and excised [7]. Prompt surgical management of the malformation is critical to preserve and possibly improve neurological function in up to 95% of cases [5].

## 5. Conclusion

In summary, the resulting neurological deficits which resulted from an acute infection of the dermal sinus illustrate the potential risks of a spina bifida occulta, a condition generally accepted as a benign abnormality. Additionally, the delayed imaging and surgical treatment represent the arduous circumstances of lacking financial capability to afford medical care in low-income countries.

## Figures and Tables

**Table 1:** FBP results. N: normal, L: low, H: high, Hb: haemoglobin, HCT: hematocrit, MCV: Mean Corpuscular Volume, MCH: Mean Corpuscular Haemoglobin, MCHC: Mean Corpuscular Haemoglobin Concentration, RDW: Red (Blood Cell) Distribution Width, and ESR: Erythrocyte Sedimentation Rate.

Parameter	Value	Range
Erythrocyte count	5.03 × 10^12^/L	N
Hb	11.1 g/dL	L
HCT	37.9%	N
MCV	75.4 fl	L
MCH	22.1	L
MCHC	29.3 g/dL	L
RDW	23.7%	H
Platelet count	598 × 10^9^/L	H
Leukocyte count	11.50 × 10^9^/L	H
Neutrophils	18.4%	N
Lymphocytes	70.6%	H
Monocytes	6.2%	N
Eosinophils	0.3%	N
Basophils	4.6%	H
ESR	90 mm/1 hr	H
